# U. S. V. M. A.

**Published:** 1897-03

**Authors:** 


### U. S. V. M. A.

### NOW FOR NASHVILLE.

THE National Saddle-horse Breeders' Association will offer special cups for prizes at the Centennial Exposition of Nashville, and a strong exhibit is promised.

The famous breeding-farms of Nashville will be the source of much in-erest and pleasure to visiting veterinarians after convention days are over.

A generous and kindly spirit was shown by the Kansas City veterinarians in the canvass of their representatives in the interest of Nashville.

The work of Messrs. Rayen, Dalrymple, and Cary seems to have been well done at Buffalo.

Ex-Secretary Turner, now of Indianapolis, loomed up as a strong advocate of Nashville, and polled his ardent wishes with the Executive Committee.

Of the first twelve votes of the Executive Committee, ten were for Nashville for first choice, one for Columbus, one for Kansas City; but it is to be said that both of these latter were for Nashville on second choice. Well done, Nashville!

Already we hear of the ladies preparing for Nashville, and there will be some new faces there of those who heard from Buffalo after the convention days were over. The more the merrier.

